# Supplementary material for: Compensation-Like Temperature and Spin-Flip Switch in Strained Thulium Iron Garnet Thin Films: Tuning Sublattice Interactions for Ferrimagnetic Spintronics†
Source: ACS Appl Nano Mater. 2025 Jul 10;8(29):14567–75. doi: 10.1021/acsanm.5c02082 (PMC12305490; doi:10.1021/acsanm.5c02082)
Supplement: Supplementary file 1 [file an5c02082_si_001.pdf]

Supporting Information

Compensation-Like Temperature and Spin-Flip Switch in Strained Thulium Iron Garnet Thin Films: Tuning Sublattice Interactions for Ferrimagnetic Spintronics

Carlos C. Soares,[*,†,‡,¶] Thiago J. A. Mori,[‡] Fanny Béron,[¶] Jagadeesh S. Moodera,[§,‖] Júlio C. Cezar,[‡] Jeovani Brandão,[*,‡] and Gilvânia Vilela[*,†,§]

†*Física de Materiais, Escola Politécnica de Pernambuco, Universidade de Pernambuco, Recife, Pernambuco 50720-001, Brazil*

‡*Laboratório Nacional de Luz Síncrotron, Centro Nacional de Pesquisa em Energia e Materiais, 13083-970, Campinas, SP, Brazil*

¶*Instituto de Física Gleb Wataghin, Universidade Estadual de Campinas, Campinas SP, 13083-859, Brazil*

§*Plasma Science and Fusion Center, and Francis Bitter Magnet Laboratory, Massachusetts Institute of Technology, Cambridge, MA 02139, USA*

‖*Department of Physics, Massachusetts Institute of Technology, Cambridge, Massachusetts 02139, USA*

E-mail: carlos.soares@lnls.br; jeovani.brandao@lnls.br; gilvania.vilela@upe.br

Phone: +55 (21)99525-2535

Discussion on Sum Rule Analysis

To gain deeper insights into the magnetic interactions and sublattice dynamics in the TmIG thin film, X-ray absorption spectroscopy (XAS) and X-ray magnetic circular dichroism

(XMCD) measurements were conducted. These techniques provide site-specific information about the electronic and magnetic properties of the ions, enabling a detailed examination of the Fe and Tm sublattices and their contributions to the overall magnetic behavior.

The orbital and spin angular moments for both Fe and Tm were determined by applying sum rules to the integrated XAS and XMCD spectra.[1,2] These calculations are based on the relationship between the integrated dichroic signals and the electronic states involved in the transitions. To apply the sum rules to Fe and Tm, three different integrals must be calculated: p, the XMCD integral over the L_3 (or M_5) edge; q, the XMCD integral over the full absorption spectrum; and r, the XAS integral after background subtraction. For Fe, the background was estimated using a standard double-step function. In the case of Tm $M_{4,5}$ edges, a third-degree polynomial was first used to remove the spectral background, followed by the application of a double-step function to isolate the XAS edge structure. These procedures ensure accurate estimation of the integrals required for the sum rule analysis.

The sum rule equations are given by:

$$\langle S_{\text{eff}} \rangle = m_s = 2\langle S_z \rangle + 7\langle T_z \rangle = -\left(\frac{6p - 4q}{r}\right) N_h \tag{S1}$$

$$m_l = \langle L_z \rangle = -\left(\frac{4q}{3r}\right) N_h \tag{S2}$$

for Fe $L_{2,3}$ edges, corresponding to $2p \rightarrow 3d$ transitions, and

$$\langle S_{\text{eff}} \rangle = m_s = 2\langle S_z \rangle + 6\langle T_z \rangle = -\left(\frac{5p - 3q}{r}\right) N_h \tag{S3}$$

$$m_l = \langle L_z \rangle = -\left(\frac{2q}{r}\right) N_h \tag{S4}$$

for Tm $M_{4,5}$ edges, associated to $3d \rightarrow 4f$ transitions.

Here, r represents the integrated total XAS intensity at the absorption edges, and p (q)

correspond to the integrated XMCD intensities at the L_3/M_5 (L_2/M_4) edges. The terms $\langle L_z \rangle$, $\langle S_z \rangle$, and $\langle T_z \rangle$ denote the orbital angular momentum, spin angular momentum, and magnetic dipole operator components, respectively.

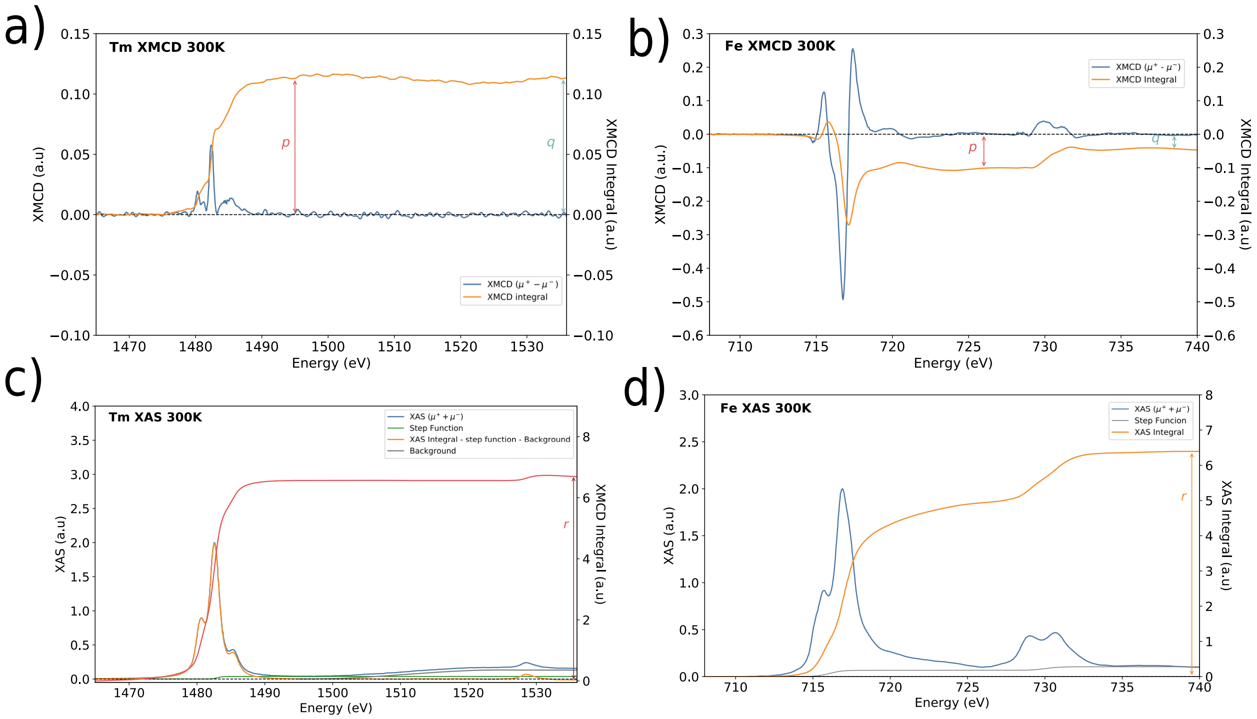

Figure S1: XMCD spectra and total XMCD integral of (a) Tm edge and (b) Fe edge at 300 K of TmIG film. XAS spectra, total XAS integral, step function, and background subtraction of (c) Tm edge and (d) Fe edge at 300 K of TmIG film.

The number of holes in the valence shell (N_h) is taken as 4.7 for Fe ($3d^5$) and 2 for Tm ($4f^{12}$).[3] This adjustment reflects the electronic configuration of Tm^{3+} in the TmIG lattice, where 12 electrons occupy the $4f$ orbitals, leaving $N_h = 2$ as the number of unoccupied states. The term $\langle T_z \rangle$ represents the magnetic dipole operator, which accounts for interactions between the spin moment and the anisotropic charge distribution. This term is critical for systems with strong spin-orbit coupling, such as the $4f$ orbitals of Tm. In contrast, for Fe, which primarily derives its magnetism from the $3d$ orbitals, $\langle T_z \rangle$ is often neglected due to the relatively isotropic charge distribution in the cubic symmetry environment of the Fe ions.[3–5] Error bars for the spin and orbital moments were estimated by varying the integration limits of parameters p, q, and r within a reasonable range. Errors related to the number of holes and

spin-orbit mixing were considered negligible compared to those from integral estimations.

For Fe, we assume $\langle T_z \rangle \approx 0$, allowing us to determine the expectation value of the spin operator $\langle S_z \rangle$ directly from the spin sum rules. However, for Tm, the strong spin-orbit coupling and anisotropic charge distribution require explicit consideration of $\langle T_z \rangle$. In this case, we use the ratio $\langle T_z \rangle / \langle S_z \rangle$ for Tm^{3+}, obtained from free-ion calculations.[6] This assumption is valid as long as the crystal field perturbation remains smaller than the spin-orbit interaction, which generally holds for lanthanide elements.

By incorporating this ratio into the spin sum rule for Tm, the equation simplifies to:

$$\langle S_z \rangle = -\frac{\frac{(5p-3q)N_h}{r}}{2 + 6\left(\frac{\langle T_z \rangle_{\text{free}}}{\langle S_z \rangle_{\text{free}}}\right)} \tag{S5}$$

References

(1) Carra, P.; Thole, B. T.; Altarelli, M.; Wang, X. X-ray circular dichroism and local magnetic fields. *Phys. Rev. Lett.* **1993**, *70*, 694–697.

(2) Chen, C. T.; Idzerda, Y. U.; Lin, H.-J.; Smith, N. V.; Meigs, G.; Chaban, E.; Ho, G. H.; Pellegrin, E.; Sette, F. Experimental Confirmation of the X-Ray Magnetic Circular Dichroism Sum Rules for Iron and Cobalt. *Phys. Rev. Lett.* **1995**, *75*, 152–155.

(3) Tripathi, S. XMCD Investigation at $M_{4,5}$ Edges of the Rare Earth Elements in High-Performance Permanent Magnets. Doctor of Natural Sciences (Dr. rer. nat.), University of Stuttgart, Stuttgart, Germany, 2018; Supervised by PD Dr. Eberhard Goering and Prof. Dr. Jörg Wrachtrup.

(4) Omar, G. J.; Gargiani, P.; Valvidares, M.; Lim, Z. S.; Prakash, S.; Suraj, T. S.; Ghosh, A.; Lim, S. T.; Lourembam, J.; Ariando, A. Room Temperature Strong Orbital Moments in Perpendicularly Magnetized Magnetic Insulator. *Adv. Funct. Mater.* **2025**, *35*, 2414188.

(5) Vasili, H. B.; Casals, B.; Cichelero, R.; Macià, F.; Geshev, J.; Gargiani, P.; Valvidares, M.; Herrero-Martin, J.; Pellegrin, E.; Fontcuberta, J.; Herranz, G. Direct observation of multivalent states and $4f \rightarrow 3d$ charge transfer in Ce-doped yttrium iron garnet thin films. *Phys. Rev. B* **2017**, *96*, 014433.

(6) Teramura, Y.; Tanaka, A.; Thole, B.; Jo, T. Effect of Coulomb Interaction on the X-Ray Magnetic Circular Dichroism Spin Sum Rule in Rare Earths. *J. Phys. Soc. Jpn.* **1996**, *65*, 3056–3059, Journal SEP VK272 J PHYS SOC JPN.
